# Care with child development and André Bullinger’s special look at prematurity

**DOI:** 10.1590/1984-0462/2022/40/2020416

**Published:** 2022-04-04

**Authors:** Mônica Regina da Silva Raiol, Sylvie Viaux Savelon, Marcia Maria dos Santos de Moraes

**Affiliations:** a Universidade Paris 7, Paris, França.; b Pitié Salpêtrière, Paris, França.; c Universidade Federal do Sul da Bahia, Teixeira de Freitas, BA, BraZil.

**Keywords:** Infant, premature, Child development, Psychomotor performance, Neonatology, Prematuridade, Desenvolvimento infantil, Desempenho sensório-motor, Neonatologia

## Abstract

**Objective:**

To assess different ways of caring for preterm infants’ development and for their families in neonatal units, with emphasis on the studies by André Bullinger.

**Data source:**

A review of the literature in the databases PubMed, SciELO, and the Cairn.info portal, which publishes reviews in human sciences in French. Also, the books and articles of André Bullinger, available only in French, were reviewed.

**Data synthesis:**

This review includes the Kangaroo Method, which is based on skin to skin contact and the encouragement of breastfeeding; the Newborn Individualized Developmental Care and Assessment Program (NIDCAP), based on the Synaptic Developmental Theory and aiming to positively change the neonatal environment, having the preterm newborn as the actor of their own development and the mother as a regulator; and the Bullinger Approach, which uses a sensory-motor perspective to approach child development, including preterm infants’ development.

**Conclusions:**

The Kangaroo Method has changed child developmental care in countries with limited financial resources. NIDCAP was shown to be efficient, although only a few long-term studies have been conducted on the subject. The Bullinger Approach is well diffused in European neonatal units, with promising results for the prevention of neurodevelopmental disabilities, especially those related to orality

## Resumo

**Objetivo:**

Abordar diferentes formas de cuidado em relação ao desenvolvimento de crianças prematuras e suas famílias nas unidades neonatais, com ênfase nos estudos de André Bullinger.

**Fontes de dados:**

Revisão da literatura nas bases de dados PubMed e Biblioteca Eletrônica Científica Online (SciELO), no portal Cairn.info, que publica revisões em ciências humanas e sociais de língua francesa, e nas obras de André Bullinger, disponíveis somente na língua francesa.

**Síntese dos dados:**

Esta revisão contempla o Método Canguru, que é centrado no contato pele a pele e no incentivo ao aleitamento materno; o Newborn Individualized Developmental Care and Assessment Program (NIDCAP), que se baseia na teoria sináptica do desenvolvimento e visa modificar positivamente o ambiente neonatal, sendo o prematuro ator do seu próprio desenvolvimento e sua mãe a reguladora; e a Abordagem Bullinger, que tem um olhar para o desenvolvimento da criança numa perspectiva sensório-motora, com trabalhos dedicados à prematuridade.

**Conclusões:**

O Método Canguru transformou os cuidados com o desenvolvimento infantil em países com recursos financeiros limitados. O NIDCAP tem se mostrado eficaz, apesar de poucos estudos em longo prazo. A Abordagem Bullinger, muito difundida e mais utilizada nas unidades neonatais da Europa, tem resultados promissores na prevenção de patologias do neurodesenvolvimento, especialmente no que tange aos distúrbios da oralidade.

## INTRODUCTION

According to the World Health Organization (WHO), around 15 million babies are born prematurely in the world each year.^1^ Recently, due to the progress in obstetrics and perinatal intensive care, the prognosis of these infants became promising; however, extreme prematurity, that is, birth before 28 weeks of gestation, is associated with an increased risk of long-term pathologies and complications.^2^

According to the Epipage 2 survey—an epidemiological, prospective cohort study carried out in 25 regions of France—, around 60,000 preterm infants are born in the country per year, that is, 180 per day, of which 1.2% are born before 32 weeks of gestation. This number has increased in recent years. The partial results of the follow-up of these children at 2 years of age show a high frequency of neurodevelopmental disorders, especially among those born at 24–34 weeks of gestation.^3,4^

In Brazil, currently, 340,000 preterm infants are born each year—11.7% of the total number of births, which represents 931 preterm infants per day. Among these, 60–70% develop normally and about 10–15% are at risk for serious disabilities such as cerebral palsy, severe intellectual disability, blindness, and deafness.^1^

From 30 to 50% of moderate preterm newborns have a type of disorder and can face consequences in education and adulthood. Environmental, socioeconomic and familial factors often influence the long-term development of these children.^5^ The neonatal environment is usually noisy, bright and with numerous tactile stimuli. Although essential for the survival of babies, this environment can impact negatively on their development, especially on orality.^6^

This paper addresses the Kangaroo Method, the Newborn Individualized Developmental Care and Assessment Program (NIDCAP) and the Bullinger Approach, as they are the most widely used forms of care aiming at the development of preterm infants worldwide in neonatal units; however, emphasis is placed on the studies by Bullinger with the aim of spreading this knowledge.

## FORMS OF CARE AIMED TO THE DEVELOPMENT OF PRETERM INFANTS

### The Kangaroo Method

The Kangaroo Method was introduced with the works of dr. Sanabria, in 1978, and today it is the most used method in developing countries. It consists of keeping the baby directly on the mother’s womb, in skin-to-skin contact, contributing to the well-being and health of the newborn, and helping in thermal regulation and emotional bonding with the mother.^7^

Studies show that using this method can reduce the incidence of nosocomial infections,^8^ help maintain thermal and cardiorespiratory stability, bring more mature sleep organization, and benefit the newborn’s neurobehavioral development.^9^ Exclusive breastfeeding and duration of breastfeeding rates are higher in children who are exposed this method.^10,11^

The Kangaroo Method has been recommended in Brazil since 1999 and takes place in stages. The first step takes place in preterm-risk pregnancy and continues in the Neonatal Intensive Care Unit (NICU). The second one takes place at the Kangaroo Unit, where the mother is constantly with her newborn. After hospital discharge, the third stage begins, when the child is followed up by the hospital and primary care teams.^12^

A basic premise of this method is to provide a harmonious and global development of preterm infants in the motor, affective and sensory domains. Thus, it proposes strategies to reduce stress during neonatal care, encourages the presence of parents in care and assesses the indications, recommendations and benefits of the kangaroo position for the baby and their parents.^12^

## NEWBORN INDIVIDUALIZED DEVELOPMENTAL CARE AND ASSESSMENT PROGRAM

Als et al., creators of the NIDCAP and the Assessment of Preterm Infants’ Behavior (APIB) scale,^13^ developed the synaptic theory of development, describing five major subsystems present in infants that overlap and must be in harmony in order to provide balance. These subsystems are: autonomous, motor, state (deep sleep-wake), attention, and self-regulation. According to the authors, these subsystems act in cascade over each other. Thus, a baby who has insufficient periods of sleep will be disorganized in the motor sphere, at risk of having apnea and bradycardia, in addition to hardly being able to sustain their look and interact with their parents. The NIDCAP is based on the principle of preterm infants being the actor of their own development, while the mother is their natural regulator and the health professionals are the supporters.^14,15^

The purpose of NIDCAP is to avoid unexpected sensory loads and pain and to emphasize the newborn’s positive aspects and skills. This program adapts the intensive care and the environment to the individuality of the child’s neurodevelopment, thus responding to their clinical and developmental needs. Care is adapted to support the positive points of each baby and their self-regulation, through the collaboration of their parents, who should be primarily responsible for daily care and for the construction of the affective bond.^14^

A review of randomized controlled trials and effectiveness of interventions showed positive results of the NIDCAP in neonatal units, despite insufficient data to assess medium-term benefits.^16^

## BULLINGER’S SENSORY-MOTOR APPROACH

André Bullinger, a child-development psychologist, created a way of caring for children known as developmental care. It is both a sensorimotor approach and, more than a method, a philosophy of care.^17^

Bullinger coined the concept of sensorimotor care based mainly on the work of Jean Piaget, who coined the term “sensorimotor” to describe the period of development that goes from birth to 24 months of life. For Piaget, motor and sensory stimuli will feed the child’s psychic domain, and they will adapt their body and elaborate the first knowledge to optimize psychomotor development.^17^

## THEORETICAL BACKGROUND

For Bullinger, space is not an object in the environment; it is the result of coordination and its components qualify it. Therefore, child development follows an order of different spaces that precede each other: the uterine space, gravity space, oral space, chest space, trunk space, and body space.

The uterine space is in fact a stimulant and tactile exchange between the back of the fetus and the uterine wall. The fetal movements respond to contractions of the uterine muscle, which put the baby into a curled-up position. It is a dialogue between the fetus and the mother.

The gravity space is the coordination between vestibular signals and signals coming from tactile sensations, muscle supports and fores, as well as tendon and bone forces. This space is the essential foundation for postural shaping.

The oral space, already present in intrauterine life, is the first in which instrumental functions such as sucking and swallowing manifest. Tactile and olfactory flows participate in this instrumental activity. The balance between these components is necessary so that this space is a fulcrum that will facilitate the development sequence.

The chest space (*espace du buste*) corresponds to the balance between flexion and extension. Inside the uterus, fetal movements are supported by the uterine wall, which contains the fetus’ extensions and ensures its flexion. After birth, it is the human environment that must compensate for this balance through an adequate way of holding the baby that allows exchanges with the environment through the creation of a dorsal support and the discovery of a ventral plane. These elements are necessary for the balance between extension and curling, after which the left and right coordinations are established.

**Figure 1.**
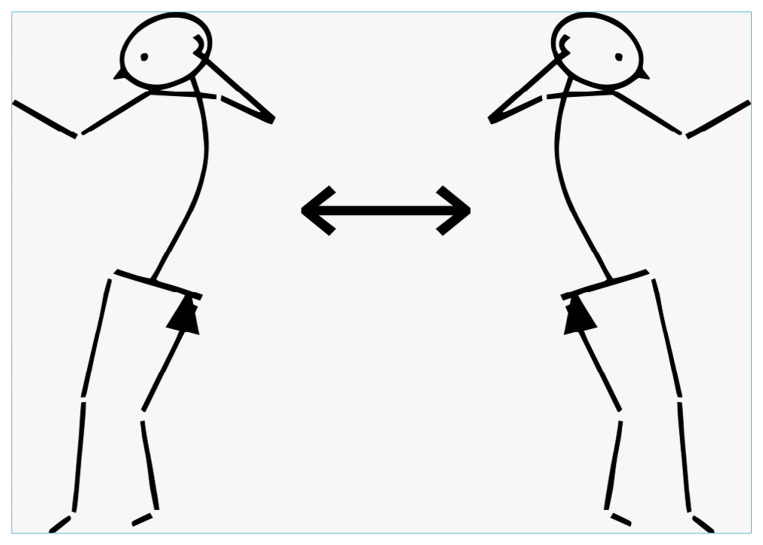
Right and left asymmetrical positions described by André Bullinger.^17^

Trunk space (*espace du torse*) begins when the upright position of the trunk is achieved by balancing two asymmetrical postures (Figure 1). At this point in development, and as a result of coordination, the child is able to turn their head, hold an object in both hands, and explore right-left coordination. When this coordination is established, the mouth is freed from much of its exploration functions and can invest in vocal games, prefiguring language.

The space of the body begins with the verticalization of the chest and the dissociation of the scapular and pelvic girdles, allowing the beginning of the rolling of the hips. At that moment, the child, lying on the back, manages to catch their feet, and this mobilization of the hip has repercussions in different domains. It is a key step in psychomotor development. The possibility of rolling the hip leads to a differentiated tonic regulation of the lower limbs and, when this connection between the upper and lower parts of the body is performed, the legs provide a set of signals that will contribute to the beginning of gait.

Thus, sensorimotor development represents a sequence of steps that fit together to achieve mastery of body spaces. It is the successive experience of these different spaces that contributes to the construction of the body axis, linking postural acquisitions, sensorimotor coordination and spatial notions. The body axis appears not simply as postural support, but also as both representative and emotional support.^17^

## PRACTICAL BACKGROUND

Each sensory system is composed of archaic and recent systems. The archaic system discriminates qualitative aspects, such as pleasant/unpleasant sensations, temperatures, textures and movements, ensuring a tonic-emotional response. The recent system corresponds to learning and is related to quantitative aspects. When the archaic system (primitive reflexes) dominates, it can give rise to different forms of prehension or irritability if the balance with the recent system does not happen.^17^

In prematurity, environmental conditions are disturbing, and the sensorimotor balance is disordered. In the incubator, for example, the baby may have inadequate stimulation or even lack of them, preventing interactions with the environment and the maintenance of a psychic activity.^18^

The positioning of the body axis can be compromised by the lack of “curling up” when little support is offered. So, in order to fully accompany the newborns in the different spaces described by Bullinger, the oral area should, in principle, be stimulated to preserve the active role of food and, therefore, of pleasure, in addition to maintaining the coordination between the right and left spaces of the body. At the same time, the chest space must be prepared for the curl by the supports. These notions of sensorimotor balance, sensory flow and positioning show the need to adapt the environment, invest in oral domain, and support parenthood.^18^

### Environmental adaptations

#### Positioning

**Figure 2.**
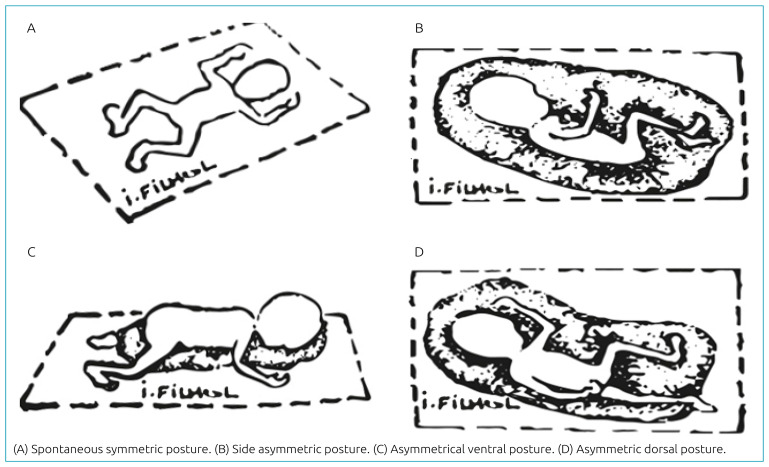
Spontaneous symmetric posture and asymmetric postures described by Martinet.^19^

At birth, the baby loses the continence they had inside the uterus, when they had their back pressed against the uterine wall and responded with extension movements to the variations in tactile, auditory, vestibular and olfactory sensory flows, which allowed for a tonic dialogue with their mother. When birth is premature, due to hypotonia and muscle weakness, the baby cannot fight the force of gravity or put themselves back into the physiological curled up position they were in the uterus, then the symmetrical posture predominates (Figure 2A). In this spontaneous position, the preterm infant keeps the arms in a candlestick position and the legs in a frog-like position, with hyperextension of the nape and upper chest prevailing, which blocks spontaneous motricity and exposes them to risks. Without resources to return to their physiological position, the human environment is then responsible for recreating an adequate posture.^17^

Asymmetrical positioning is essential to favor the sensorimotor development of preterm and sick children, and may have variations such as: right/left lateral, slightly lateralized ventral and dorsal positions, as shown in Figures 2B, 2C and 2D. Each of these postures brings a specific comfort for breathing or digestion, enabling spontaneous motricity, avoiding retractions, improving swallowing, oculo-manual coordination and mouth exploration.^19^

To facilitate the asymmetrical posture, long pillows must be used to form a circle, like a nest, in which the baby is placed keeping the head in alignment with the hip. This posture favors the division and alternation of the right and left sides of the body, in addition to slightly improving the body axis, which can support the head. Thus, the infant will have wakefulness increased and will be able to turn to objects in their environment.^19^

This adapted positioning ensures a containment effect, delimiting a space materialized by the nest. The baby, thus contained and sustained, stabilizes more quickly if any event disorganizes them.^18^

#### Sound and visual adaptations

With regard to sound adaptations, parents are encouraged to talk to their children through the incubator door, so that they can locate the sound source and identify the speaker’s voice. Professionals should not place objects on the incubator or speak loudly near babies, in order to limit resonance effects. It is not about suppressing environment sound, but making the environment meaningful and able to participate in the baby’s sensory integration.^18^

As for the visual adaptations, the incubator walls convey a blurry image of the environment that does not favor the tonic-postural reactions normally raised by the peripheral vision, which is part of the archaic system and, thus, is present since birth. The newborn’s eye movements are sudden and irregular, seeking to maximize cortical excitations. The eye is stimulated by light fluctuations, and the gaze is drawn to chromatic contrast. The attraction to contrasted shapes is what encourages pediatricians to use a target made of white and black circles, to facilitate eye movement and help the baby to follow an object. This practice helps to stabilize the sensory-tonic balance, ensuring optimal interactions between the child and the environment they are in.^18^

### Oral investment to prevent eating disorders

One of the conditions for the baby to go home is the acquisition of active feeding from the breast or from a bottle. Lack of coordination between breathing-suction and swallowing before 33–34 weeks of gestation is compensated for by tube feeding for preterm infants. But in this situation, the child receives more food than they can digest, and the term “gavage” illustrates well this idea of passivity. In this regard, it is important to outlaw the use of this term, which evokes forced eating and gives an inhumane idea of eating. Thus, the term “passive feeding” seems more appropriate.^18^

This way of administering diet deprives babies of intense sensorimotor experiences in terms of taste, odor, consistency, temperature, and texture, in addition to limiting the use of sensory and motor skills of the mouth, particularly of the tongue, lips, gums, and the palate.^19^

Feeding requires coordination between gripping and exploration, as well as oral and tactile coordination of objects—a balance between recent and archaic tactile signals.^20^

The feeding activity takes place in a narrative dimension, like a story that will be a source of pleasure if told well. The different elements taken into account to achieve globality are developed according to the chronology described by Bullinger:^20^ posture + smell + suction + swallowing + satiety = hedonic aspects.

The quality of holding the baby, the exchanges of looks and verbalizations constitute a narrative that gives meaning to this sequence of events. Once the infant is well positioned and sensitized by the maternal odors they rediscover, the bonding can occur followed by exploration of the nipple or bottle by the tongue, allowing the start of sucking. After feeding, the feeling of satiety will be valued by the family circle and the baby, containing and contained, will feel the first representation of continence: a differentiation between the inside and the outside.^20^

The establishment of food is complex and involves numerous factors that cannot be reduced to the action of eating. In fact, these steps help the infant to have a good relationship with feeding and, if difficulties occur, they should be explored. A balance between these different components is necessary so that the oral space is constituted and becomes a point of support for the upcoming stages of development.^20,21^

#### Description of perioral and oral stimuli

Currently, the term “request to stimulation” is preferred, as it expresses the attention that should be given to the baby’s responses. The technique used to stimulate the perioral and oral spheres is based on the sensory and biomechanical components of the oral area, described by Weber in 1852, showing that the region close to the ear lobe is the least sensitive area of the cheek, while the labial commissure is the most sensitive area. This perioral stimulus, while respecting the sensitivity gradient, gives the infant the possibility to accept or refuse the stimuli without a tonic hyperextension response, which could disorganize the posture required when receiving food.^17^

The Neonatology Service of the University Hospitals of Geneva recommends a protocol of oral stimuli that maintain and/or rehabilitate the oral activity in infants on tube or parenteral feeding. Such guidelines, described below, favor a spontaneous grasp of the object that reaches the mouth, prevents an invasive contact and offers olfactory and gustatory stimuli.^19,23,24^

#### Stimuli protocol

- To pass a cotton swab over the cheeks, starting from the ear lobe to the labial commissure.
- To observe the lip orientation movements during stimulus. If an immediate response is not given, restart the stimulus three or four times on each side.
- To soak the cotton swab with milk (breast milk, if available) and pass it first around the lips and then over them.
- If the child opens their mouth, pass the swab soaked with milk (breast milk, if available) over the gums, gingival crypts, inside cheeks, and hard palate.
- Once the child puts the tongue in the suckling position, let them suck on the cotton swab. Tube or bottle feeding can then be started.
- If the child is fed by tube, allow them to suck their thumb, fingers or pacifier while feeding through the tube.

### Parenting support

Preterm newborns have particular needs and their parents discover them differently, each in their own way. Therefore, the care team must adapt the environment to allow parents continuous access to intensive care units. This provides longer contact time, supports breastfeeding and encourages participation in care, allowing parents to be partners in their children’s development. The barriers constituted by medical care and procedures, in addition to the physical separation imposed between them, reinforce the need for psychological support in this period.^25^

Studies show that the presence of parents has a positive impact on brain plasticity, development and functioning, minimizing motor, cognition and behavioral disorders.^26^

## FINAL CONSIDERATIONS

There are several ways to approach psychomotor development in childhood, including the Kangaroo Method, the NIDCAP and the Bullinger Approach. The latter considers the sensory-motor period as the first stage of life, when the child organizes until the building of their body. In his last works, Bullinger dedicated to the care of preterm infants.

The Kangaroo Method has transformed the neonatal reality in several countries in a humanized and an emotional way, improving parent-infant relationship, and, from a technical point of view, by reducing infections and neonatal and infant mortality, also allowing for early hospital discharge.^26,27^

The NIDCAP has shown positive results for preterm newborns and their relatives, and for professional awareness of the importance of this care.^16,28^

Kloeckner, in his experience in neonatology, showed that the impact of the Bullinger Approach in neonatal units can be seen in different areas: the service is calmer, babies cry less, and caregivers find a decrease in gastroesophageal reflux and plagiocephaly. On the parental side, seeing their babies in a comfortable position, similar to the position in the womb, make them calm and, thus construction of parenting and interactions with their newborns can be stimulated. The author concludes that, despite the complex theory, the practice of this approach can prevent neuromotor, language, eating and parent-infant relationship disorders.^18^

With regard to the rules of dietary transitions, Bullinger’s contribution allows the infant to be an actor in their own care, as parents find their baby more quickly in a nutrition activity that weaves the paths of attachment, with caregivers as their guides. In addition, reducing hospital stay is also an important aspect when it comes to preterm infants.^24^ The Bullinger Approach may not need significant financial investments, but it does require awareness and training of professionals in this area, especially those involved with prematurity.

The Kangaroo Method’s strong point is the skin-to-skin contact, which contributes mainly to thermal control and parent-infant bonding, demonstrating the importance of this practice in this care. However, other approaches must be included, such as Bullinger’s view about birth, which considered it not as a trauma, but a sensory revolution, giving meaning to the sequence of stages of development. His special view on orality has established feeding as a sequence of components that go beyond sucking and swallowing.

The Bullinger Approach has shown the benefits of oral stimuli when performed from birth and the importance of using the nest not only as an element of containment and comfort, but also as a fundamental support in the different stages of child development.

Appropriate positioning makes it possible to consider the body shape as fundamental points of support for a proper conduct of eating, which is not simply a physiological need, but also an affective and emotional moment, as Bullinger said.

Thus, it is healthy and urgent to expand this knowledge, as well as the practice of caring for preterm infants recommended by the Bullinger Approach, so one can reduce the risk of neurodevelopmental disorders in these children.

This review also points to the need for more research on the subject and studies with a prospective-longitudinal design that could benefit newborns, particularly preterm ones.
